# Point-of-Care Nucleic Acid Detection: From Molecular Design to Clinical Reality

**DOI:** 10.1021/acsomega.5c02914

**Published:** 2025-07-03

**Authors:** Marta Marchesini, Maria Laura Costantino, Lillo Raia, Nina Bono, Gabriele Candiani

**Affiliations:** † Department of Chemistry, Materials and Chemical Engineering “G. Natta”, 18981 Politecnico di Milano, Via Mancinelli 7, Milan 20131 Italy; ‡ STMicroelectronics S.r.l., Agrate Brianza, Monza Brianza 20864 Italy

## Abstract

Point-of-Care (PoC) diagnostics are transforming healthcare by enabling rapid and accessible disease detection right at the patient’s bedside. This comprehensive review examines recent advances in nucleic acid (NA)-based PoC testing, revealing how these technologies are revolutionizing molecular diagnostics. Here, we critically analyze the three key components of NA-based PoC development: (i) probe design strategies, (ii) immobilization techniques, and (iii) detection methodologies. Our analysis uncovers the complex relationship between probe density, hybridization conditions, and detection sensitivity, challenging the conventional trial-and-error approaches currently dominating the field. The review introduces a novel classification of detection methods based on equipment requirements, offering valuable insights for developing truly accessible diagnostic solutions. Notably, we highlight that colorimetric and electrochemical detection methods show superior potential in meeting the REASSURED criteria (Affordable, Sensitive, Specific, User-friendly, Rapid and Robust, Equipment-free, Real-time connectivity, Ease of specimen collection, and Deliverable to end-users). These criteria are essential for global healthcare implementation. Despite major advances, significant gaps remain between laboratory innovations and practical, affordable diagnostic products. We suggest that prioritizing equipment-free detection methods and enhancing standardization could accelerate the translation of these PoC technologies into clinical practice. This review outlines a strategic roadmap for advancing NA-based PoC diagnostics, emphasizing the potential to transform healthcare delivery worldwide through accessible, rapid, and reliable molecular testing.

## Graphical abstract

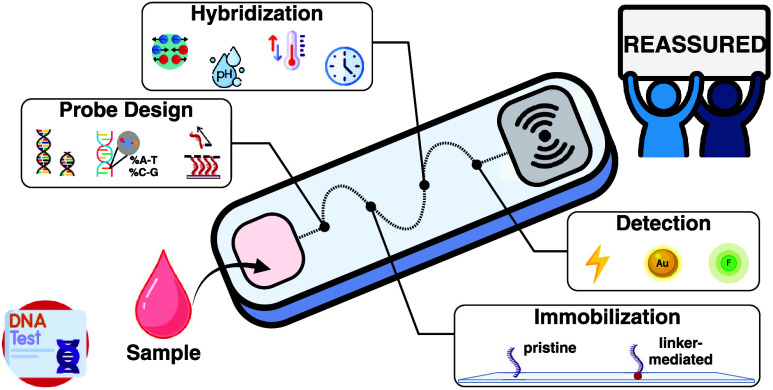

## 1 Introduction

Medical diagnostic technologies (MedTechs) are crucial tools in healthcare, facilitating disease identification and enhancing clinical decision-making.^1,2^ Traditional MedTechs primarily fall into two categories (Figure 1), such as imaging and molecular diagnostics.^3^ The latter encompasses a spectrum of laboratory technologies used to analyze molecular constituents, including DNA, RNA, and proteins, extracted from tissue samples or fluids to detect diseases.^4^ Depending on the specific target molecules under investigation, various types of molecular diagnostic tests can be employed. For example, to analyze specific fragments in a complex pool of nucleic acids (NAs), techniques such as polymerase chain reactions (PCR) are used.^5^ Alternatively, Fluorescence *In Situ* Hybridization (FISH) enables the visualization of genes (copy number and localization) or chromosomes of specific DNA targets within cells and tissues through fluorescence microscopy.^6^ Conversely, to address the detection of proteins, one can capitalize on the selective binding between antigens and antibodies to detect and localize them in cell and tissue samples. These technologies include Enzyme-Linked ImmunoSorbent Assay (ELISA)^7^ to process liquid samples, ImmunoHistoChemistry (IHC)^8^ for fixed tissues, and ImmunoCytoChemistry (ICC)^9^ for isolated cells.

**Figure 1:**
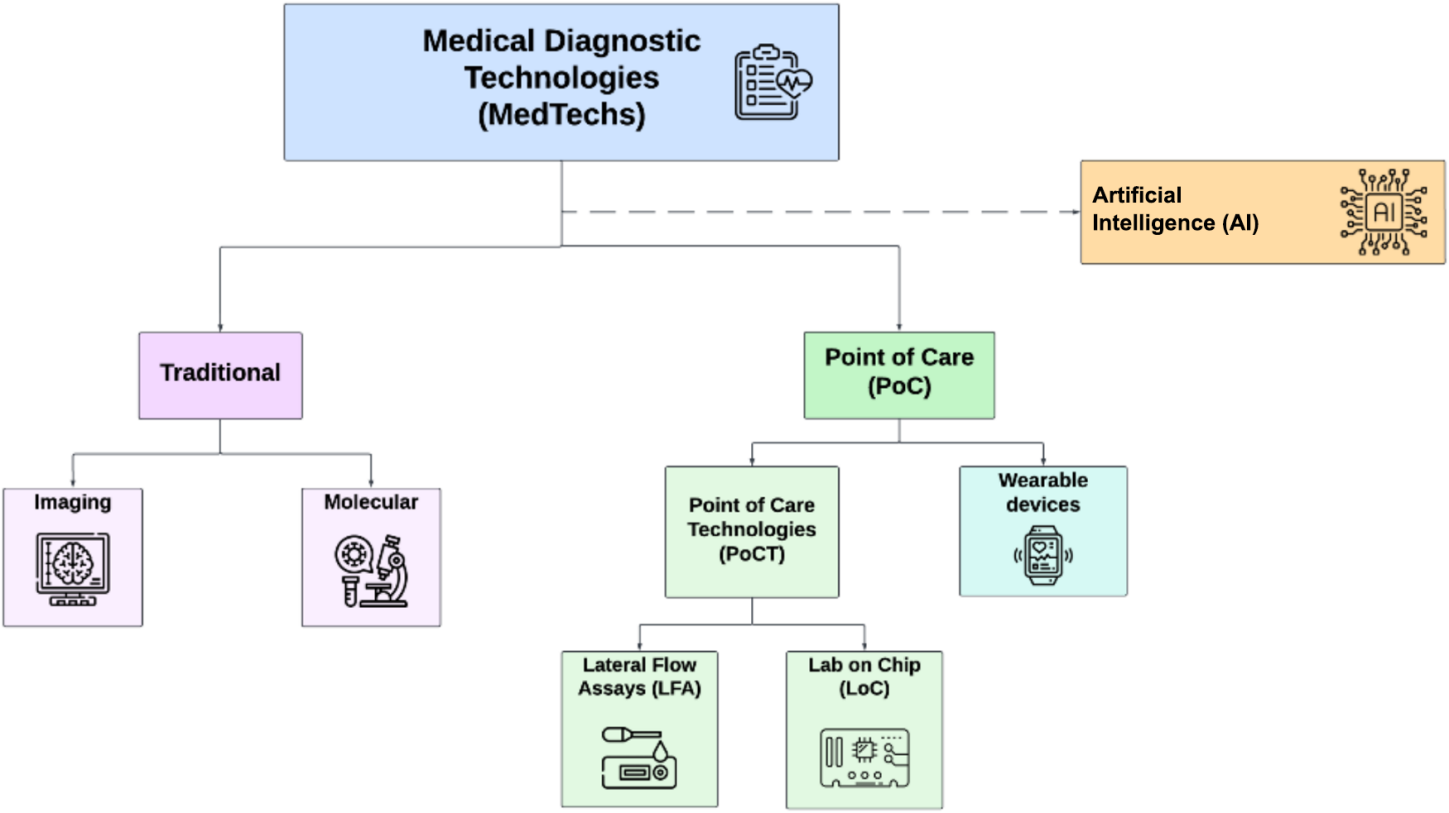
Schematic visualization of medical diagnostic technologies classification.

Traditional diagnostic methods face significant limitations, including expensive equipment, time-consuming procedures, and the need for highly skilled personnel. However, recent technological advancements are transforming the field. Innovations such as portable sensors, Point-of-Care (PoC) technologies, and artificial intelligence (AI) applications^10^ are addressing these challenges, and are revolutionizing healthcare. AI, in particular, has become increasingly important for the management and interpretation of complex diagnostic data sets. Together, these technologies provide more precise, accessible, and efficient diagnostic solutions that are reshaping modern healthcare delivery.^10^

PoC diagnostics include either PoC Testing (PoCT),^11^ or wearable devices.^12^ These diagnostics, including microfluidic devices and wearable technologies, enable early disease detection, preventive measures, and continuous health monitoring.^11^ According to the most recent market analysis, the global PoC diagnostics market was valued between 31–52 billion USD in 2023, with projected growth to 70–90 billion USD in 2030.^13,14^ This robust growth trajectory reflects increasing demand for rapid, accessible diagnostic solutions.

### 1.1 PoCT

PoC diagnostics involve conducting tests directly at healthcare delivery sites, requiring minimal infrastructure and training. These devices are designed to be fast, flexible, and adaptable across various settings—from clinics and ambulances to patients’ homes. The World Health Organization (WHO) initially established the ASSURED criteria to define ideal characteristics for PoC devices: Affordable (i.e., cost-effective for resource-limited settings), Sensitive (i.e., low false negative rates), Specific (i.e., low false positive rates), User-friendly (i.e., simple procedures that requires minimal training), Rapid and Robust (i.e., fast results within 15–60 min that withstand storage/transport conditions in terms of temperature, humidity, time delays and mechanical stresses), Equipment-free (i.e., no special instrumentation required), and Deliverable to end-users (i.e., effective distribution systems for resource-constrained settings).^15^ In 2019, Land et al.^16^ expanded the existing framework to include the REASSURED by adding two essential requirements: Real-time connectivity, which refers to the ability to transmit and analyze test results electronically for centralized diagnosis and surveillance, and Ease of specimen collection, which involves using noninvasive sampling methods that require minimal processing steps at the PoC.^13,17^ However, complying with REASSURED criteria is still hardly feasible.^18^

The typical workflow for a PoCT comprises three essential steps performed in proximity to the patient: sample collection and device loading, biomarker analysis, and result interpretation (Figure 2). While offering lower diagnostic costs and faster results, these devices can potentially reduce hospital admissions and medical expenses, though sophisticated test cartridges. The key advantage lies in their ability to provide fast, accessible diagnostic information while requiring minimal laboratory infrastructure.

**Figure 2:**
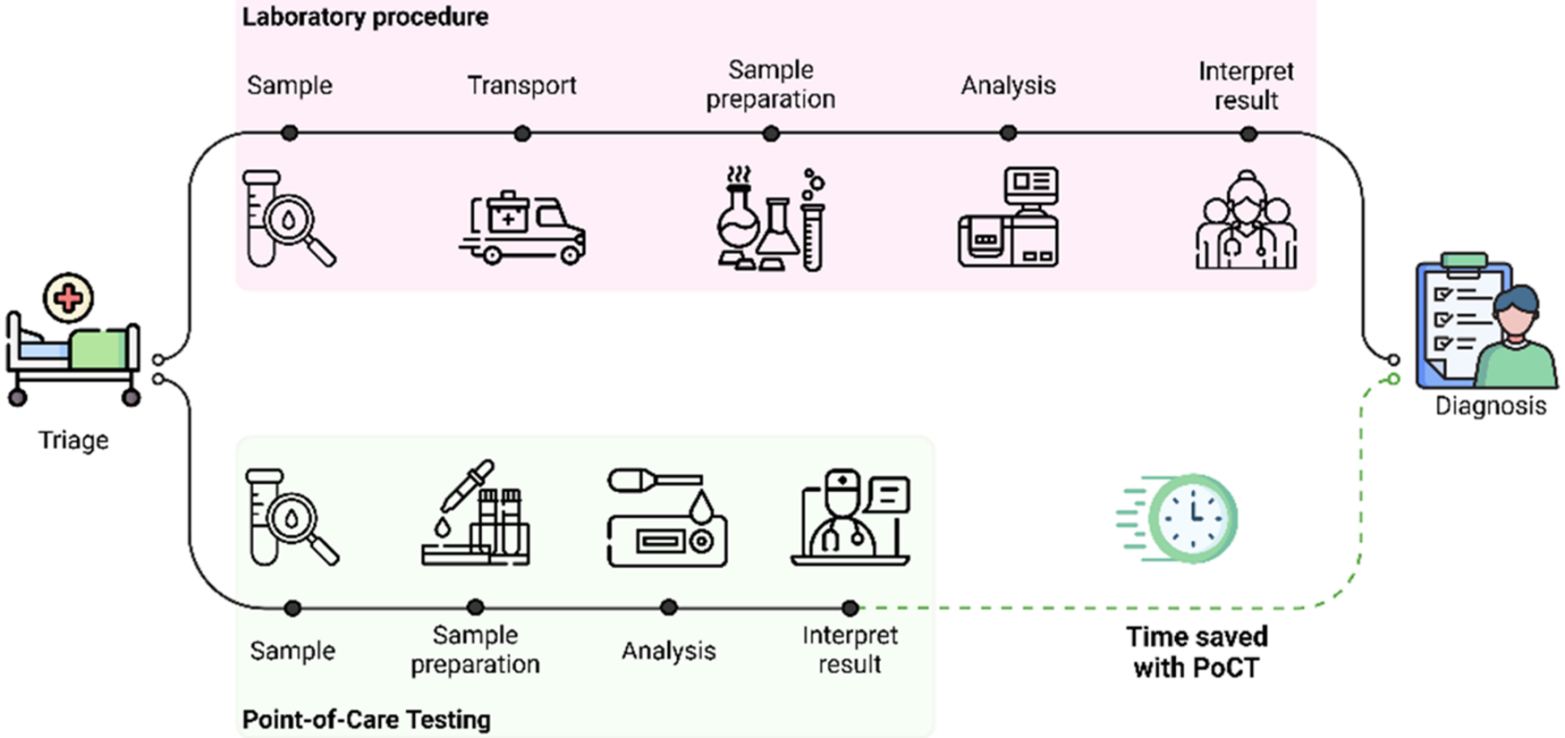
Workflow of a PoCT with respect to a conventional laboratory procedure.

PoCT devices primarily utilize two key technologies: Lateral Flow Assays (LFAs)^19,20^ and Lab-on-Chip technology (LoC).^21,22^ LFAs consist of porous strips containing embedded reagents that enable rapid protein detection through antibody–antigen interactions. These tests excel in providing simple, fast, and cost-effective diagnostics.^23^ In contrast, LoC technology integrates multiple laboratory functions onto a single microchip platform, handling everything from sample preparation to analysis. While more sophisticated in capabilities, LoC devices can be more complex and costly to develop.^21,22^ However, they minimize contamination risks and reduce dependence on specialized equipment and trained personnel.^20^

### 1.2 Nucleic Acid–Based Assays

Current PoCTs rarely satisfy all REASSURED criteria simultaneously, suggesting “assays through a platform” as a more precise descriptor of these technologies. This broader classification encompasses both LoC and paper-based tests, all designed to detect biological markers (biomarkers).^24^ These platforms primarily target two categories of biomarkers: proteins (e.g., enzymes or antibodies)^25^ and NAs (e.g., DNA, mRNA, and microRNA—miRNA).^26^ NA biomarkers are particularly valuable for their ability to specifically detect genetic mutations, viral and bacterial infections, and molecular signatures associated with physiological alterations during disease progression.

NA-based assays offer promises for rapid and reliable PoC diagnostics due to their high specificity, thermal stability, ease of modification, multiplexing capability, and reduced cross-reactivity.^27^ The specificity of these assays stems from the specific binding of complementary NA sequences, enabling accurate detection of genetic markers while minimizing false positives.^24,28^ Easily synthesized and modified, NA probes can be tailored for enhanced affinity and selectivity, adapting to various diagnostic needs.^29^ Their modular nature allows integration with diverse detection and amplification strategies, improving analytical sensitivity and accuracy. Furthermore, NA molecules exhibit high thermal stability, maintaining their structural integrity even at elevated temperatures,^30,31^ a critical attribute for ensuring reliable performance across variable environmental conditions encountered during storage, transportation, and testing. Moreover, the multiplexing capability of NA-based assays facilitates the simultaneous detection of multiple targets, crucial for complex diagnostics.^32^ Compared to protein-based assays, NA-based assays exhibit lower cross-reactivity due to the higher binding specificity during hybridization.

This review explores the current state of the art of NA-based assays, detailing their components in relation to critical PoCT criteria, including cost-effectiveness, user-friendliness, accessibility, and rapidity. We explore NA probes, with particular emphasis on immobilization and hybridization via Watson–Crick base pairing. Furthermore, we evaluate various detection systems, biosensors, and microarrays, critically assessing their suitability as PoCTs. Finally, we discuss future directions to translate promising laboratory innovations into practical, accessible diagnostic solutions for real-world applications.

## 2 Critical Design and Development of a NA-Based PoCT

The key steps in designing a NA-based PoCT include probe immobilization, sample collection with subsequent NA extraction, amplification when necessary, hybridization between probes and target sequences, and signal detection (Figure 3).

**Figure 3:**
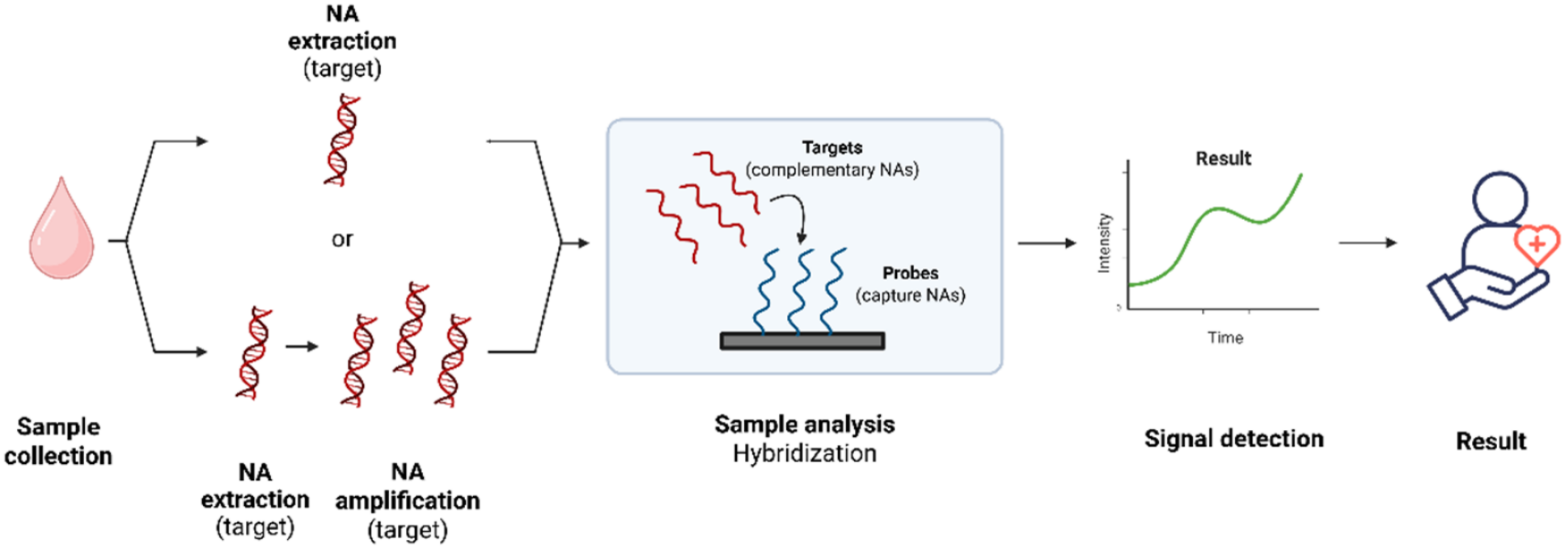
Schematic representation of general NAs detection PoCT workflow.

Although NA extraction from complex biological samples remains a major challenge in translating lab assays to PoCT^33,34^ (comprehensively addressed by Paul et al.)^33^, our review will focus on key aspects of NA-based assays: electing appropriate NA probes for targets, choosing immobilization methods, and optimizing hybridization and detection processes. Together, these processes determine overall analytical performances.

Numerous NA-based PoCT platforms have moved from laboratory research to widespread clinical use. However, a critical analysis reveals a persistent reliance on NA amplification strategies to achieve clinically relevant sensitivity thresholds.^34,35^ The Cepheid GeneXpert system features automated sample preparation combined with real-time PCR and thermal cycling within single-use cartridges. This system provides diagnostic results within 30 min and demonstrates 100% positive predictive agreement for influenza detection.^36,37^ Similarly, the Roche Cobas Liat offers a turnaround time of 20 min from sample to result, with sensitivities exceeding 99% and specificities surpassing 97% for influenza detection using integrated RT-PCR.^38,39^ Additionally, the Abbott ID NOW system delivers results within 15 min by employing isothermal amplification technology. While this platform’s speed is comparable to conventional PCR assays, certain sensitivity issues have been documented for specific targets.^40,41^ Another noteworthy example is the BioFire FilmArray platform, which allows for the simultaneous detection of 20 respiratory pathogens in 1–2 h through nested PCR multiplexing technology.^42,43^ Notwithstanding these technological advances, all aforementioned platforms require complex auxiliary equipment, strict temperature control, and intricate fluidic architectures for handling reagents and managing thermal cycling. In contrast, amplification-independent detection methodologies are still limited in the commercial PoCT landscape. Most nonamplified technologies remain confined to research settings or applications involving high-abundance analytes. This reliance on amplification-based systems reveals a significant gap in the field and highlights the critical need for optimized NA-based PoCT platforms that can comply with the ASSURED criteria without depending on complex amplification protocols.

### 2.1 NA Probe Design

NA-based assays use single-stranded (ss) DNA/RNA probes that bind to complementary target sequences through the hybridization process, following Watson–Crick base pairing rules. This binding event generates detectable optical or electrical signals that form the basis of the diagnostic readout.^44^ Probe design is crucial for effective PoCT, with probes typically being shorter ssNA sequences than their targets to facilitate rapid hybridization while maintaining high specificity. Several synthetic probe types exist.^19^ OligoNucleotides (ONs) are synthetic DNA or RNA sequences up to 50 nucleotides (nt) long. For instance, molecular beacons (MBs) use stem-and-loop ssDNA structures for mismatch detection.^45^ RNA-based probes (cRNA or riboprobes) are ssRNAs that offer high sensitivity and greater stability than DNA-RNA hybrids, while miRNAs show resistance to RNase and harsh conditions.^46^ Moreover, Peptide Nucleic Acids (PNAs), first introduced by Nielsen et al. in 1991,^47^ are synthetic DNA analogs with the anionic deoxyribose-phosphate backbone replaced by a pseudopeptide with enhanced binding affinity due to its uncharged behavior.^48,49^ For multiplexed detection, careful probe design is essential to avoid secondary structures that could interfere with target binding.^50^ Online tools like VectorBuilder’s DNA secondary structure predictor (https://en.vectorbuilder.com/tool/dna-secondary-structure.html) can help assess potential structural issues.

### 2.2 NA Probe Immobilization

NA probe immobilization is a critical step in developing NA-based assays. The process must ensure probe accessibility for efficient target binding, preserve proper orientation stability, and prevent nonspecific interactions while maintaining firm anchoring to avoid probe detachment during analysis. These factors directly influence the biosensor’s analytical sensitivity and selectivity.^51^

The selection of an appropriate immobilization technique depends on both physicochemical properties of the substrate surface and the specific characteristics of the probe molecules (DNA-based ON, RNA-based ON or PNA). Three main strategies have emerged as predominant approaches: (i) physical adsorption (physisorption), (ii) covalent immobilization (chemisorption), and (iii) avidin (or streptavidin)-biotin interaction (Table 1).^19,32^ Each method offers distinct advantages and limitations that must be carefully evaluated.

**Table 1:** Advantages and Limitations of NA ProbeiImmobilization Strategies

immobilization	binding nature	advantages	limitations	refs
physical adsorption	ionic interactions	simple and fast process	limited control over probe orientation and surface density	^52–55^
		minimal reagents and modification steps required	risk of probe detachment	
			nonspecific interactions	
			poor reproducibility	
covalent immobilization	covalent bonds	good probe accessibility	complex chemistry and modification steps required	^48,56–63^
		high binding strength		
		low nonspecific adsorption	expensive and slow process	
		long-term storage	complexity	
avidin- (or streptavidin-) biotin interaction	specific interaction between avidin (streptavidin) and biotin	strong and stable immobilization	modification steps required	^64–66^
		high specificity	expensive and slow process	
		reversible		

#### 2.2.1 Physical Adsorption

Physisorption is the simplest yet straightforward NA probe immobilization method, relying on noncovalent, ionic interactions between negatively charged NA probes and platform surfaces.^51^ PoCT substrates are typically modified with cationic polymers like chitosan, polyaniline, or polypyrrole to enhance electrostatic attraction and probe stability.^52–54^ While straightforward, as demonstrated in Ribeiro et al.’s work using chitosan-functionalized electrodes, this method has significant limitations.^55^ These include nonspecific adsorption, random probe orientation affecting hybridization efficiency, and weak binding that may lead to probe detachment due to environmental factors such as temperature, ionic strength, and pH.^51^

#### 2.2.2 Chemical Immobilization

Covalent immobilization provides significantly enhanced stability for NA probe attachment compared to physisorption while enabling better precise probe orientation for optimal hybridization.^67^ One main approach is chemisorption, where thiol­(-SH)-modified NA probes form Self-Assembled Monolayers (SAMs) on gold (Au) surfaces through gold and sulfur (Au–S) bonds.^19,68^ While widely used in electrochemical biosensors, as shown in Ananthanawat et al.’s work with HS-PNA probes,^48^ the exact nature of this bond remains debated.^69,70^ Historically, it has been assumed that hydrogen dissociates during the chemisorption process as the bond forms between Au and S. However, significant uncertainty persists regarding the fate of the thiol hydrogen during adsorption.^70–72^ Inpek et al.^71^ demonstrated that the interaction between Au and S occurs through physisorption rather than chemisorption, which has contributed to a better understanding of the key properties of Au–S SAMs. Nevertheless, the Au–S bond continues to be widely exploited in the production of electrochemical NA biosensors.^56,57,73^ Surface blocking agents such as mercaptohexanol (MCH)^74^ or Bovine Serum Albumin (BSA)^75^ can help reduce nonspecific binding.

Alternative covalent approaches involve chemical modification of surfaces and/or NA probes. Surfaces can be functionalized through silanization^58–60^ or chemically modified with linkers such as carbodiimide reagents (*N*-hydroxysuccinimide (NHS) and 1-ethyl-3-(3-dimethylaminopropyl) carbodiimide (EDC)) to enable binding of amine (−NH_2_)-terminated NAs.^61–63^ For example, Wang et al.^76^ used glutaraldehyde to link the NH_2_-tethered ON probes to chitosan-modified carbon nanotubes. While covalent binding offers advantages in specificity and stability,^60,61^ these strategies require greater chemical expertise and can increase process complexity and cost.

#### 2.2.3 Avidin/Streptavidin–Biotin Interaction

The avidin/streptavidin–biotin interaction offers the strongest noncovalent interaction, providing a highly stable method for NA probe immobilization. This approach utilizes biotin’s remarkably strong affinity (*K*_a_ = 1 × 10^–15^ M) for avidin/streptavidin proteins, which contain up to four bonding sites per molecule. The resulting interactions demonstrate extraordinary resistance to pH, temperature, exposition to organic solvents and/or other denaturing agents.^77,78^ Consequently, this method has been widely used in NA-based assays.^64–66^ Wang et al.^79^ employed this strategy in an LFA biosensor, using streptavidin–biotin bonds to immobilize DNA-based ON capture probes on the test and control lines. While providing strong attachment, this method requires probe biotinylation, typically at the 3′- or 5′-end, and surface modification with avidin/streptavidin, increasing process complexity and costs.^19^

#### 2.2.4 New Classification

The traditional classification of NA probe immobilization methods has limitations when examined through the lens of IUPAC definitions. According to IUPAC, physical adsorption refers to the process in which weak interaction forces, such as van der Waals forces or intermolecular forces (20–40 kJ mol^–1^), are involved, and the species do not significantly alter their electronic orbital patterns. In contrast, chemical adsorption occurs when the adsorbent and the adsorbate form a stronger bond (80–240 kJ mol^–1^). Additionally, IUPAC’s Gold Book defines chemisorption as a synonym for chemical adsorption; however, common use of chemisorption is to describe the chemical immobilization through Au–S binding,^68^ despite uncertainties about the binding mechanism and associated bond energy. Additionally, the strong avidin/streptavidin–biotin interaction could technically qualify as chemical adsorption under IUPAC definitions.

In this context, we propose a new classification system based on immobilization requirements rather than theoretical binding mechanisms: (i) pristine immobilization, which means direct and straightforward surface adsorption of molecules, and (ii) linker-mediated immobilization, which requires modification of NA probes and/or surfaces. For instance, chitosan-functionalized electrodes by Ribeiro et al.^55^ represent surface linker-mediated immobilization, while Au–S binding is an example of NA probe linker-mediated immobilization.^48,68,73^ Some methods, like Leonardi’s SARS-CoV-2 biosensor using (3-Glycidyloxypropyl)­trimethoxysilane (GOPS)-modified silicon nanowires (NWs) with amino-modified NA probes, require modifications of both surface and probe.^80^

### 2.3 Probe-Target Hybridization

Hybridization occurs when complementary ssON (DNA/RNA) form double-stranded assemblies through Watson–Crick base pairing. In PoCT assays, specific target sequences bind to their complementary probes, with several critical factors affecting the process. Target NA concentration must be carefully considered; excessive target concentrations can cause nonspecific binding and elevated background noise, while low concentrations must still exceed the system’s detection thresholds target NA concentration for reliable measurement. Environmental conditions, such as temperature, pH, salt concentration, buffer composition, and hybridization duration, must be optimized to balance sensitivity with assay turnaround time.^81,82^

To shed light on the influence of these parameters, we computed a correlation matrix analysis of literature data. As clearly shown in Figure 4, there is no strong correlations between these parameters and the Limit Of Detection (LOD)—defined as the lowest concentration of the target that can be consistently detected. This lack of evident correlations suggests that current approaches are predominantly empirical and trial-and-error based. To advance PoCT diagnostic technologies, more rigorous and scientific methods are needed. Design of Experiments (DoE) approaches, which systematically analyze how multiple input factors affect desired outputs, could help optimize hybridization parameters and their impact on LOD.^83^

**Figure 4:**
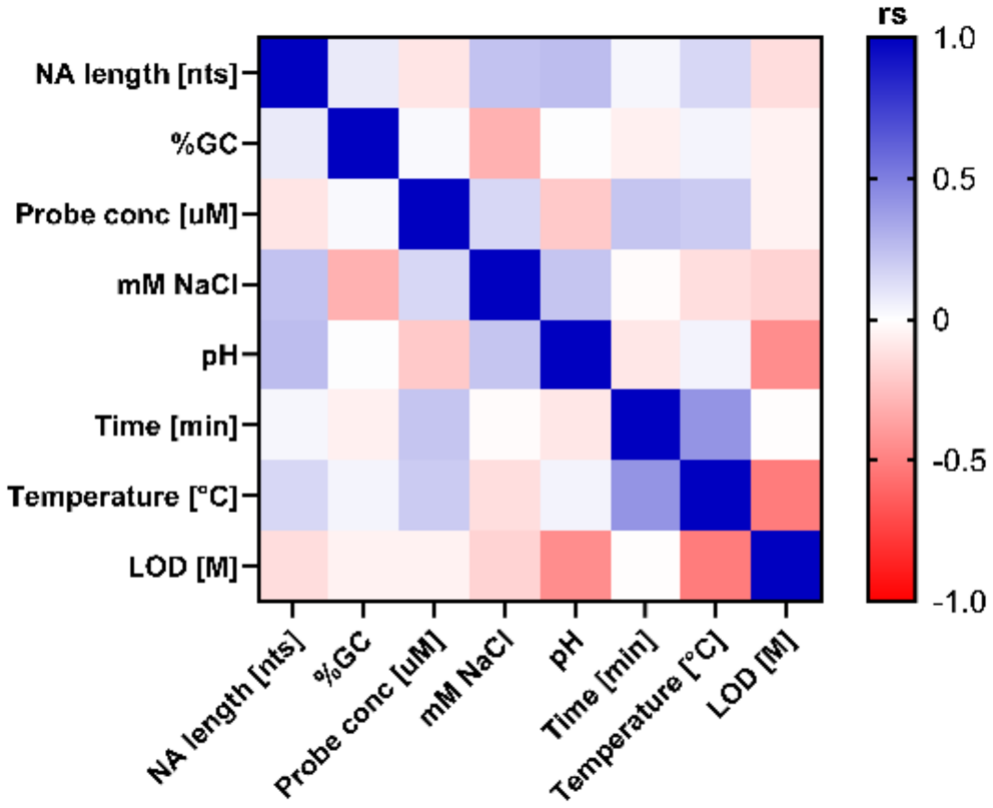
Correlation matrix of the parameters that influence the LOD of the assay. The values are color-coded with negative values in red and positive values in blue; darker shades indicate lower and higher values. The nonparametric two-tailed Spearman correlation was performed with GraphPad Prism 8.0.2. Spearman’s rank correlation coefficient (rs) ranges from −1 to +1, indicating the strength and direction of the correlation between two variables.

The following sections will examine key parameters affecting hybridization and their influence on the assay performance.

#### 2.3.1 NA Probe Density

Similar to target NA, NA probe concentration significantly affects hybridization kinetics and assay sensitivity—a crucial parameter that must be carefully optimized. Excessive probe concentrations may lead to steric hindrance that impedes target access, while insufficient density reduces binding capacity and thus sensitivity.^84,85^ Seminal studies by Peterlinz et al.^86^ identified thresholds for optimal surface coverage. Their research showed optimal hybridization occurs at approximately 5 × 10^12^ probe NA molecules/cm^2^. Specifically, when surface density remains at or below this threshold, hybridization efficiency exceeds 60%. However, when probe density increases beyond 1 × 10^13^ NA/cm^2^, hybridization efficiency drops sharply maybe because of electrostatic repulsion and steric hindrance that together prevent target molecules from effectively accessing and binding to the probes.^87^ Despite the critical importance of this parameter, the underlying molecular mechanisms governing density-dependent hybridization remain poorly understood. This knowledge gap persists largely because actual probe density is rarely directly assessed in experimental studies.^57,74,87–89^ Most literature reports only the initial probe concentrations rather than the actual surface density.

#### 2.3.2 Hybridization Temperature

Hybridization temperature (*T*_H_) is the optimal temperature at which the hybridization between NA probes and targets occurs most effectively. This parameter requires careful optimization to achieve specificity and efficiency: temperatures below the optimum can cause mispriming, self-folding, homobinding, and heteroduplex formation, while temperatures above the optimum might lead to incomplete or weak hybridization (Figure 5).

**Figure 5:**
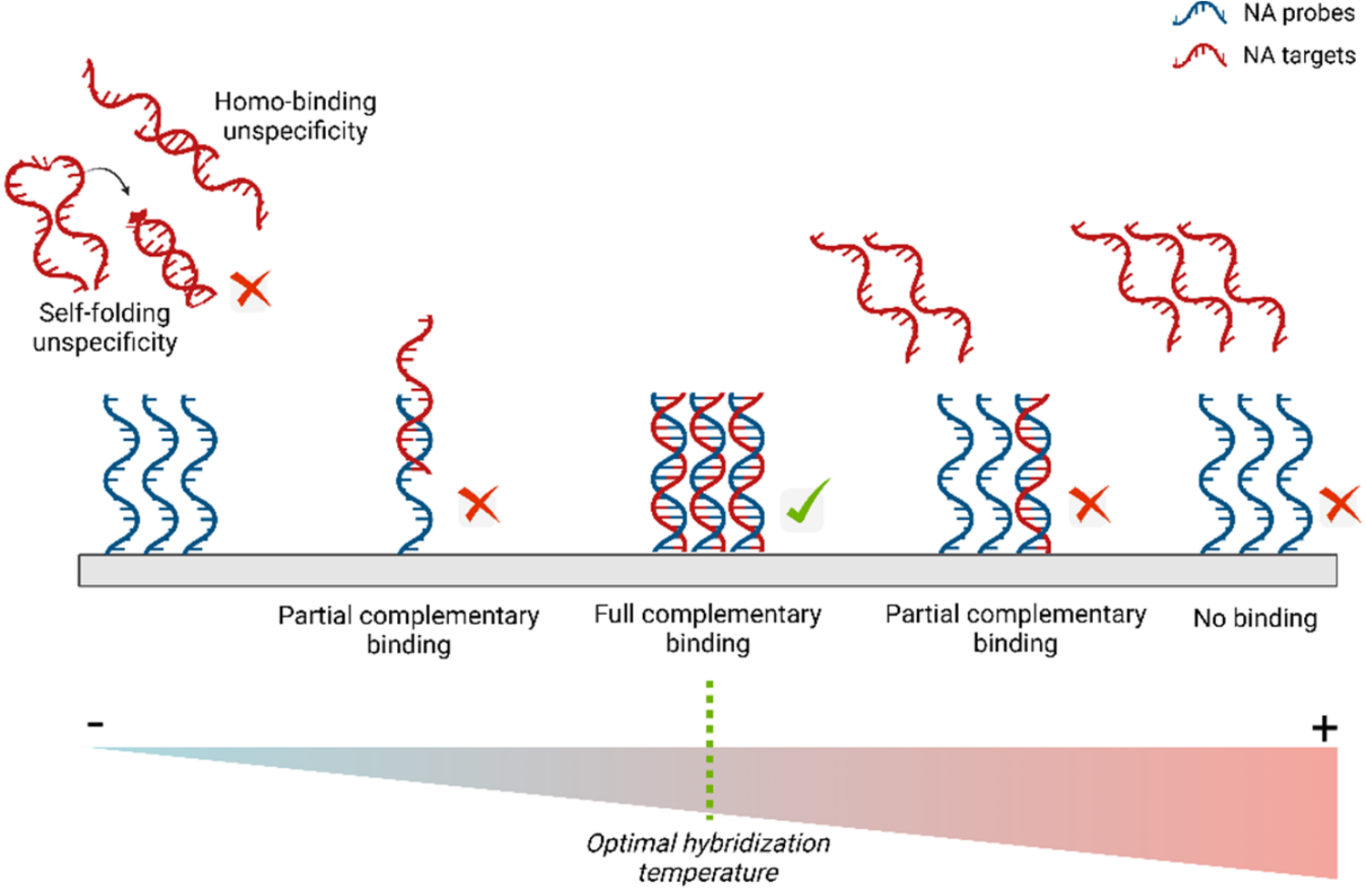
Hybridization between two ssNAs, which serve as probes and targets, results in binding interactions. Fully complementary binding is the most desirable outcome. However, unspecific binding can occur in several forms, including partially complementary binding, self-folding (hairpin formation), or homobinding (the formation of homodimers).

Temperature also affects hybridization kinetics and the formation of secondary structures within single NA sequences.^90,91^ The *T*_H_ is typically set 5–10 °C below the melting temperature (*T*_m_) of the probe-target duplex, where 50% of the duplex molecules exist in a denatured state. For short NA probes/targets, *T*_H_ usually ranges between 50–65 °C. However, the optimal *T*_H_ depends on multiple factors (Table 2), such as nt sequence length and composition (particularly GC content), probe concentration, and ionic strength.^88,92,93^

**Table 2:** Parameters that Influence *T*_H_ and Their Effect

parameter	effect
NA length	*T*_H_ increases with increasing NA length
NA base composition	*T*_H_ increases with increasing % GC content (due to triple hydrogen bonding)
NA concentration	*T*_H_ decreases with increasing NA concentration (due steric hindrance)
divalent cations	*T*_H_ decreases with increasing divalent cation concentration
ionic strength	*T*_H_ decreases with increasing ionic strength
pH	*T*_H_ lowers between pH 5 and 9

Long sequences with high GC content typically necessitate high *T*_H_, while high probe concentrations results in lower *T*_H_ as a consequence of reduced duplex stability and increased steric hindrance, particularly under conditions of low ionic strength.

Divalent cations, particularly magnesium (Mg^2+^), enhance hybridization. They, indeed, bind the negatively charged phosphate backbones of NA probes and targets, shielding them and reducing the repulsive forces, which allows the complementary sequences to approach each other more easily and form stable duplexes.^88,92,94^

Buffer pH also significantly influences hybridization efficiency, with physiological pH generally providing optimal conditions. Hybridization rates have been reported to decline significantly below pH 5 and above pH 9.^94^ Based on published data, we plotted the LOD as a function of pH (Figure 6) and identified a trend showing that as pH approaches 8, the LOD tends to decrease, indicating improved analytical sensitivity under these conditions. This observation is supported by statistical analysis showing a significant negative correlation between pH and LOD, as reported in Figure 4 (rs = −0,447, *p*-value = 0.048, *n* = 20).

**Figure 6:**
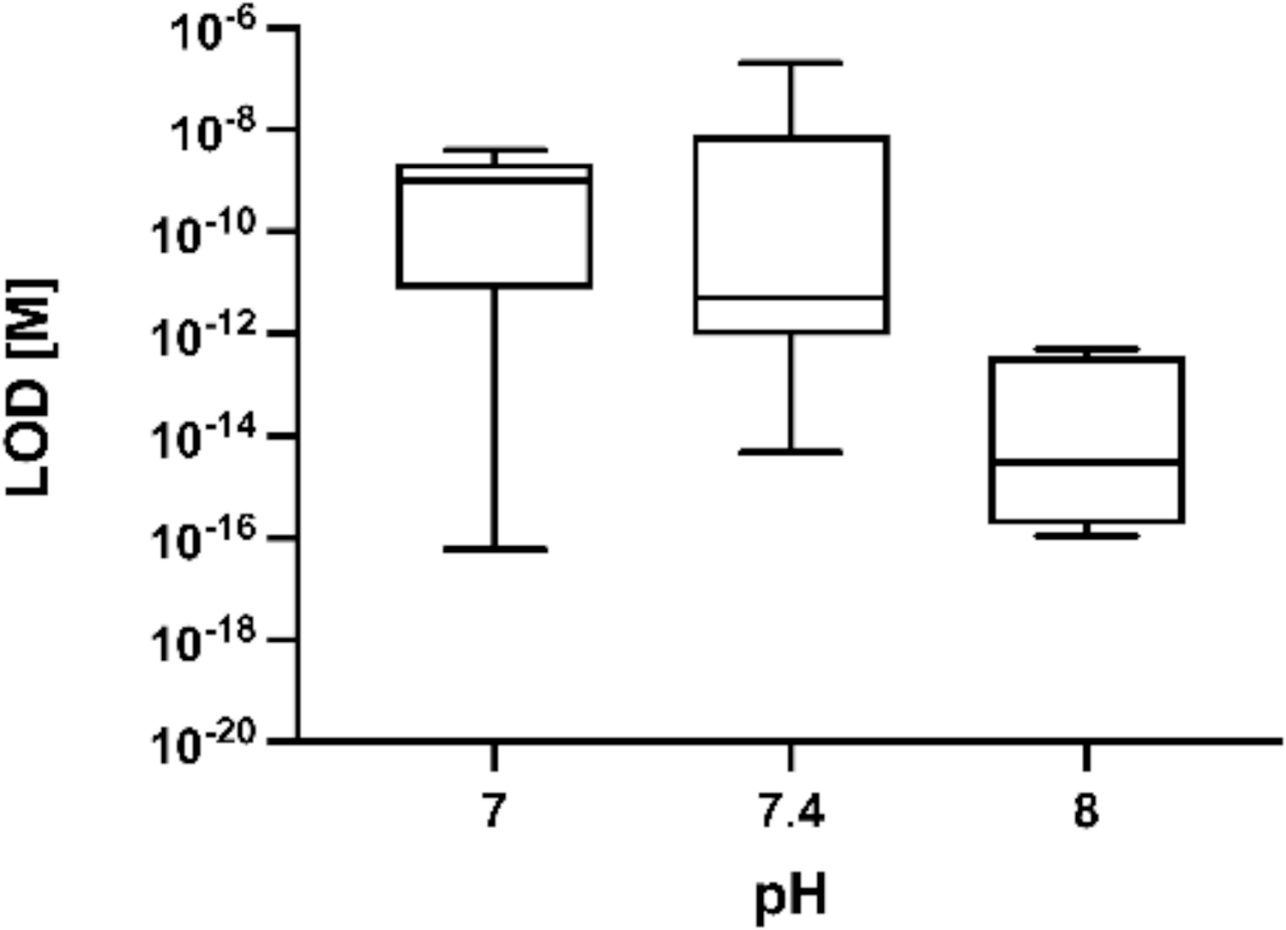
Relationship between LOD and pH. Lower and upper fence are 25th and 75th percentiles, the median is in between, and bars represent 5th and 95th percentiles.

It is worthy of note that significant temperature inconsistencies have been reported in literature. Specifically, among the papers we analyzed (*n* = 50), 38% of studies did not specify the temperature at which hybridization occurred, and 20% claimed room temperature (RT, arbitrarily assigned 25 °C) (Figure 7A). However, RT definitions vary significantly across standards and regulatory frameworks (e.g., FDA defines RT = 15–25 °C), potentially compromising experimental reproducibility. Temperature should not be treated as a constant but rather as a critical process parameter requiring precise control and documentation. Again, our correlation analysis (Figure 4) showed a significant negative relationship between temperature and LOD (rs = −0.514, *p*-value = 0.003, *n* = 31), indicating that increasing temperature leads to improved detection sensitivity of PoCTs (Figure 7B).

**Figure 7:**
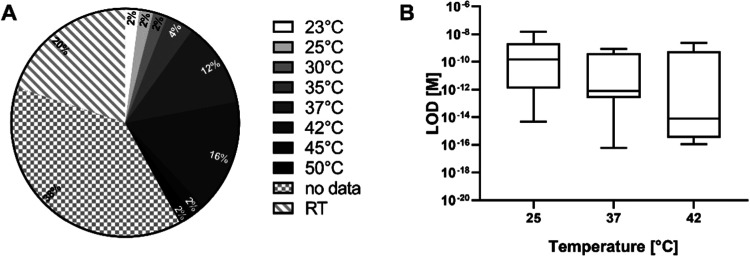
(A) Pie chart of the temperature reported in the literature. (B) The relationship between LOD and temperature. Lower and upper fence are 25th and 75th percentiles, the median is in between, and bars represent 5th and 95th percentiles.

To determine the optimal *T*_H_, researchers typically perform temperature gradient experiments using a thermocycler to identify the condition that yields the strongest signal-to-noise ratio and most specific probe-target binding.^95,96^ (Online) tools such as TMTOOLSM (https://dna-utah.org/tm/tool.php) help calculate *T*_m_ for DNA and RNA sequences based on parameters such as nt sequence, concentration, and salt content. However, these algorithms were primarily meant for PCR applications and solution-phase hybridization, lacking accuracy for solid-support systems. Experimental evidence shows that NA duplexes melt at lower temperatures on solid supports compared to liquid phases, indicating reduced duplex stability.^97^

Therefore, determining the optimal *T*_H_ requires empirical validation through systematic testing, such as evaluating signal-to-noise ratios at various temperatures around the calculated *T*_m_. Tsai et al.’s colorimetric PoCT for tuberculosis diagnosis exemplifies this systematic approach to temperature optimization.^98^

#### 2.3.3 Hybridization Time

Hybridization time in PoCT refers to the critical interval during which complementary NA sequences (probes and targets) form stable duplexes under controlled conditions. For PoCT applications, this parameter must be specifically optimized to balance analytical performance with clinical utility.

Prolonged hybridization periods are supposed to increase probe-target duplex formation, potentially enhancing assay efficiency and enabling detection of low-abundance sequences. However, extended durations are often accompanied by with the risk of nonspecific binding and elevated background noise.^82^ In contrast to liquid-phase PCR protocols, hybridization on solid support is considerably slower due to mass transport limitations. The interplay between minimal sample volumes (typically several μL) and surface interaction layer (50–100 μm) significantly impedes molecular diffusion, frequently extending the hybridization process to overnight (O.N.) durations.^99^ Evidence demonstrates a direct relationship between probe diffusion time and the analytical LOD.^100^ Consequently, optimization of the hybridization time is essential to achieve maximum sensitivity and specificity within the shortest practical time frame.

### 2.4 Hybridization Detection

Hybridization detection in PoCT refers to analytical methods that identify specific NA sequences through molecular recognition between complementary strands. The detection system must efficiently distinguish between hybridized and nonhybridized molecules. Detection encompasses three primary strategies: (i) amplification of target NA, (ii) implementation of high-sensitivity detection systems, or (iii) a combination thereof.

Amplification increases target NA concentration, reducing detection sensitivity requirements, but necessitates thermal control and enzymatic processes.^34^ Isothermal amplification techniques (e.g., Loop-mediated isothermal amplification (LAMP), Helicase-Dependent Amplification (HAD), Recombinase Polymerase Amplification (RPA), Nucleic Acid Sequence Based Amplification (NASBA)) are gaining traction.^101,102^ Alternatively, developing sensitive detection systems involves combining sample ssNA with complementary immobilized ssNA, potentially simplifying device design and reducing time-to-result.^103^ Signal transduction modalities predominantly utilize optical, electrochemical, or gravimetric signals, each with distinct advantages and limitations. Selection criteria for optimal detection strategies encompass assay-specific performance parameters, detection thresholds, sample complexity, and desired throughput. Persistent technical challenges include NA immobilization, controlling hybridization to prevent nonspecific binding, and accurate signal reading for reliable data interpretation.^104,105^

The following sections will provide a comprehensive exploration of these detection technologies in detail.

#### 2.4.1 Optical Detection

Optical detection methods rely on changes in luminescence, fluorescence, or absorbance during hybridization events. While offering high sensitivity and multiplexing capabilities, these techniques often require additional equipment that conflicts with the requirements of PoCT device.^106,107^

Fluorescence assays predominate in this category, employing a wide range of fluorophores that absorb and emit electromagnetic radiation across multiple wavelengths, spanning from ultraviolet to near-infrared regions of the spectrum. Two primary detection approaches are (i) the use of fluorescent dyes^93,108^ (intercalating dye such as SYBR Green^93^ or nonintercalating reporters including nanoparticles (NPs)) or (ii) application of molecular fluorophores as direct detection elements.^60,109^ In most cases, the target DNA can be covalently labeled with fluorophores (e.g., fluorescein isothiocyanate (FITC)^65^), enabling direct monitoring of hybridization kinetics through fluorescence measurement. Instead, MBs^45^—ONs incorporating both a fluorophore and quencher at terminal positions within a stem-loop configuration—can be immobilized on substrate surfaces. Upon hybridization with complementary target sequences, conformational changes induce spatial separation between the fluorophore and quencher, resulting in measurable fluorescence emission.^110^

Overall, the major strength of optical detection lies in its ability to minimize background noise and provide accurate, rapid detection of hybridization events.^19,111^ However, significant limitations persist, including the need for trained operators, external equipment, and high costs, which collectively impede full compliance with ASSURED criteria.^112^

Chemiluminescence in PoCT refers to an optical detection methodology where light emission results from chemical reactions occurring during NA hybridization events. Unlike fluorescence-based techniques, it does not require an external excitation source, making the detection setup simpler. This has enabled the development of rapid and automated detection techniques,^113,114^ exemplified by Mallard et al.’s DNA detection system using HorseRadish Peroxidase (HRP) and luminol oxidation, capable of detecting subpicomolar target concentrations within 30 min.^115^ Another noteworthy approach relies on the use of metallic NPs as alternatives to conventional enzymatic labels. These nanomaterials can catalyze chemiluminescent reactions with enhanced stability and reproducibility compared to their protein-based counterparts. Metallic NPs, including gold, silver, and platinum nanomaterials—demonstrate superior catalytic efficiency for reactions such as luminol oxidation while maintaining activity under broader environmental conditions.^106,107,113^ Despite the significant potential for ultrasensitive DNA detection using nanoparticle-enhanced chemiluminescence, this technique remains technically complex and time-consuming to develop.^106,107^

Colorimetric detection has emerged as a simpler, cost-effective alternative to fluorescence and chemiluminescence methods. This technique operates through detection of chromogenic changes induced by target-probe binding and can be implemented either by visual assessment (equipment-free) or spectrophotometric measurement (equipment-dependent). In PoCT, equipment-free colorimetric detection typically uses NP aggregation,^116^ antiaggregation,^117^ or etching processes.^118^ When target NAs bind to probes, they alter NP interactions, causing visible color changes. Gold NPs (AuNPs) are commonly used due to their stability and ease of functionalization, exhibiting characteristic red-to-blue chromatic shifts upon aggregation.^119^ For example, Wen et al.^120^ developed a colorimetric detection system using hairpin ON probes that assemble in the presence of target DNA, causing AuNP aggregation and color shift, achieving LOD of 0.1 pM. Similarly, Yang et al.^121^ developed a lateral flow strip for Human PapillomaVirus Type 16 (HPV16) detection using copper oxide (CuO) NPs, generating visually identifiable brown-colored detection brown bands within 20 min. While colorimetric detection offers rapid, simple, and affordable NA detection and complies with ASSURED criteria,^89^ its main limitations are qualitative analytical outputs and lower sensitivity compared to alternative detection modalities.^19,101^

#### 2.4.2 Electrochemical Detection

Electrochemical detection systems monitor NA hybridization through changes in electrical properties such as current, potential, conductance, impedance, and capacitance.^122–124^ When immobilized NA probes anchored to electrode surfaces hybridize with complementary target sequences, three principal physicochemical phenomena occur: (1) increasing of negative charge at the electrode due to NA’s negatively charged backbone; (2) conformational changes from coiled ssNA probes and targets to stretched ds-duplexes; (3) enhanced conductivity through the duplex’s π-stack, enabling electron transfer from electroactive molecules.^68,125,126^ The detection can be either label-based (indirect) or label-free (direct). In label-based detection, probes or targets are tagged with redox molecules that produce current changes upon hybridization events, offering excellent discrimination between ssNA and dsNA configurations.^119,121^ Conversely, label-free electrochemical detection has emerged as a promising alternative to labeled methods, addressing drawbacks including high costs and complex labeling.^19^ This approach exploits intrinsic electrochemical properties. Detection typically occurs through: (1) direct oxidation of nucleobases at electrode surfaces, (2) changes in interfacial electron transfer kinetics upon hybridization, or (3) alterations in electrode capacitance and impedance resulting from duplex formation. NAs are inherently electrochemically active, with guanine residues exhibiting low redox potential, enabling direct oxidation detection.^68,100^ However, this direct approach has limitations in sequence discrimination capabilities and analytical sensitivity, particularly in complex biological matrices where multiple electroactive species may coexist. To enhance signal detection, electroactive molecules (e.g., methylene blue) are often incorporated to interact with the negatively charged NA backbone.^56,110,127^ These redox-active compounds can intercalate between base pairs or bind to specific structural features of the duplex, generating amplified electrical signals upon hybridization events.

Overall, electrochemical transducers offer significant analytical advantages, including high sensitivity, low cost, and compatibility with microfabrication technologies.^128,129^ For instance, Kongsuphol’s group developed a multisensor microchip array enabling the simultaneous detection of multiple bladder cancer DNA biomarkers using porphyrin-modified probes.^130^

Despite potential compliance with ASSURED criteria and promising miniaturization, significant challenges remain, including expensive potentiostats and complex data interpretation.

#### 2.4.3 Others

Gravimetric detection systems employ Quartz Crystal Microbalance (QCM) technology to measure mass changes during NA hybridization.^131^ In QCM sensors, a piezoelectric quartz disc positioned between metal electrodes undergoes mechanical deformation under an electric field, with its resonant frequency exhibiting precise correlation with surface mass accumulation.^131^ This allows the detection of NA sequences with sufficient molecular weight (MW), typically several hundred bp-long, through hybridization-induced mass changes.^89^ While these systems offer advantages such as label-free detection, high precision, reliability, and compact size, they require targets with sufficient MW to generate detectable signals.^64,89^ Su et al. overcame this limitation using Mutator S (MutS) protein (90 kDa) that selectively binds to DNA mismatches, significantly increasing the mass on the QCM surface and enhancing the detection sensitivity of even single-nt mutations.^64^

Surface-Enhanced Raman Scattering (SERS) amplifies the optical signals produced when light interacts with NAs placed near metallic surfaces, particularly NPs in suspension or deposited onto solid substrates.^132,133^ The metal surfaces create localized electromagnetic fields that intensify the Raman signal by orders of magnitude, enabling the detection of extremely low concentrations of NAs, even in complex biological samples.^134^ Fu et al.^135^ demonstrated this approach by developing a SERS-based LFA for Human Immunodeficiency virus DNA (HIV1-DNA) using AuNPs functionalized with DNA probes. Their system created sandwich structures (capture DNA-target DNA-AuNP-probe DNA) that generated strong SERS signals, achieving a LOD of 8 pg/mL. While SERS provides powerful detection capabilities, its dependence on expensive Raman spectrometers limits its practical application in resource-limited settings.^19^

#### 2.4.4 New Classification

A practical way to classify NA detection methods for PoCT is based on whether they require equipment, which directly relates to the ASSURED criteria (Table 3). Technologies requiring external instrumentation for detection typically increase costs and need trained operators, making them less aligned with ASSURED principles of affordability, user-friendliness, and accessibility to end-users.

**Table 3:** Strengths, Weaknesses and Compliance with ASSURED Criteria of Different Detection Technologies^a^ ^b^ ^c^ ^d^

detection technology	working principle	strengths	weaknesses	compliance with	refs
Equipment-Free					
colorimetric	color development	real-time monitoring	not quantitative outcome	ASSURED	^119,121^
electrochemical labeled	changes in current, voltage, or impedance	multiplexing	NA sample modification needed	ASSRED	^96,123,146^
			buffer interference		
electrochemical label-free	changes in current, voltage, or impedance	multiplexing	buffer interference	ASSRED	^128–130^
		use of pristine NA samples			
Equipment-Required					
fluorescence	fluorescence emission	multiplexing	NA sample modification needed	SSR	^60,93,108,109,111^
		real-time monitoring	susceptibility to environmental light interference		
chemiluminescence	light emission	real-time monitoring	NA sample modification needed	SS	^113–115^
surface-enhanced Raman scattering	changes in Raman intensity	quantitative outcomes	expensive	SSR	^79,134,135^
		multiplexing			
quartz crystal microbalance	changes in mass	real-time monitoring	high MW NA probes required	SSR	^64,66^
		use of pristine NA samples			

a ASSURED = Affordable, Sensitive, Specific, User-friendly, Rapid and Robust, Equipment-free, and Deliverable to end-users.

b ASSRED = Affordable, Sensitive, Specific, Rapid and Robust, Equipment-free, and Deliverable to end-users.

c SSR = Sensitive, Specific, Rapid and Robust.

d SS = Sensitive and Specific.

Colorimetric detection represents the most successful equipment-free approach in PoCT, exemplified by widely used diagnostics such as pregnancy tests and COVID-19 rapid antigen tests. Though these established tests primarily target protein biomarkers, significant progress has been made in developing NA-based detection systems using colorimetric principles. Recent innovations include the tuberculosis detection (a bacterial infection caused by *Mycobacterium tuberculosis*) platform using AuNP aggregation to produce visible color changes detectable via a smartphone, achieving a LOD of 1.95 × 10^–2^ ng bacterial DNA/mL in 60 min.^98^ Another example is the SARS-CoV-2 that employs phenol red as a colorimetric indicator in saliva sample. This test can detect viral NAs at concentrations as low as 200 NA copies/μL in less than 60 min.^136^ These technological developments demonstrate how colorimetric detection can meet key PoC requirements, including real-time connectivity, rapidity, and affordability.^137^ Consequently, colorimetric detection currently stands as the only technique that meets all ASSURED criteria.

Electrochemical detection technology offers significant potential for PoCT applications through integration with wearables, implants, microfluidics, and/or printed circuit boards.^124^ These miniaturized systems can connect to smartphones for power, data processing, and display, meeting both equipment-free and real-time connectivity criteria.^12,113,138^ A notable example is the portable device for pancreatic cancer detection by Genco et al.,^28^ which uses single-molecule-with-a-large-transistor (SiMoT) technology with disposable cartridges and 3D-printed sensing gates, achieving a 96% sensitivity and 100% specificity in detecting both protein and NA biomarkers. However, challenges remain in making integrated electrochemical detection practical for PoC diagnostics, particularly in improving signal transduction and device miniaturization.

Fluorescence detection, despite its analytical strengths in sensitivity and specificity, faces limitations in PoC diagnostics. Although efforts have been made to develop portable fluorescence-based technologies, they remain challenging to implement.^139,140^ Current portable fluorescence systems target environmental analytes,^141^ bacteria,^142^ proteins,^143^ and ions,^144^ with NA detection systems lagging in development. These systems require complex components, including light sources, optical filters, lenses, and photon detectors.^140^ The fundamental requirement for complex optical components classifies fluorescence detection as equipment-dependent, typically requiring external instruments (microarray scanners,^58,61^ microscopes,^65,145^ or smartphone attachments^111,139,141^ ) that limit true PoC deployment.

Chemiluminescence, SERS, and QCM all require specialized external instrumentation for data collection and analysis. Specifically, a luminometer is needed for chemiluminescence,^57,114^ Raman spectrometers are used for SERS,^79,134,135^ and a piezometer is necessary for QCM.^64,66^ Additionally, chemiluminescence demands controlled storage conditions, such as refrigeration, humidity, and protection from light, to maintain the stability of enzymatic reagents and luminescent substrates. This requirement limits its applicability in PoCT. Consequently, these detection technologies are categorized as equipment-requiring, making them less suitable for true PoC applications.

### 2.5 Quality Control Implementation

The diagnostic reliability of NA-based PoCT fundamentally relies on effective quality control strategies aimed at minimizing false positives and optimizing signal-to-noise ratios, which are crucial for maintaining accuracy and specificity.^147^ To reduce false positivity, comprehensive negative control strategies are essential. These should include extraction blanks to monitor for cross-contamination during sample processing, environmental controls to detect contaminations from the surroundings, and specificity controls using closely related but nontarget sequences to confirm the selectivity of the assay.^148,149^ Optimizing the signal-to-noise ratio involves a range of complementary approaches. These include using background signal controls with blank matrices to establish baseline measurements, detection calibration controls with standardized reference analytes to normalize platform sensitivity, and environmental controls to account for external factors such as temperature fluctuations.^150^ Advanced PoCT platforms have to integrate real-time monitoring of these control parameters with automated algorithms that invalidate results if predetermined thresholds are not achieved.^151^ However, a key challenge remains: how to balance comprehensive quality control implementation with the simplicity and cost-effectiveness essential for PoC deployment, especially in resource-limited settings where technical expertise may be limited.

## 3 Biosensors and Microarray for NA Detection

A key focus in NA detection is on DNA biosensors (genosensors), and microarrays (gene/DNA chips).^126^

Biosensors function as analytical platforms that transduce biological recognition events into measurable signals.^132,152^ They combine molecular probes that interact with target analytes with physical transducers, transforming probe-target binding signals into (proportional) electrical or other outputs.^153^ The principal advantage of biosensor technology for PoC applications stems from their minimal sample preparation requirements, facilitating rapid, cost-effective analysis in nonlaboratory settings.^153^ This fueled their implementation in various fields including food analysis,^154^ bioterrorism surveillance,^155^ environmental monitoring,^156^ and healthcare diagnostics.^157^

Microarrays represent an advanced integration of multiple biosensors, enabling multiplexed analysis of numerous biomarkers simultaneously. The technology consists of tens to thousands of microscopic spots (10–100 μm-wide) containing distinct, immobilized probes (DNA, RNA, or antibodies) onto substrates such as glass, polymer, or nitrocellulose membranes. This technology has broad applications, spanning from transcriptomics, infectious disease diagnostics, antimicrobial resistance profiling, and host response characterization.^158^

Both NA biosensors and microarrays work by detecting hybridization between surface-immobilized ssON probes and complementary target sequences. The key difference lies in their arrangement: NA biosensors have probes directly on the sensor’s sensitive surface,^145,159^ while microarrays feature patterned reactive spots, each with different probe sequences^160,161^ (Figure 8).

**Figure 8:**
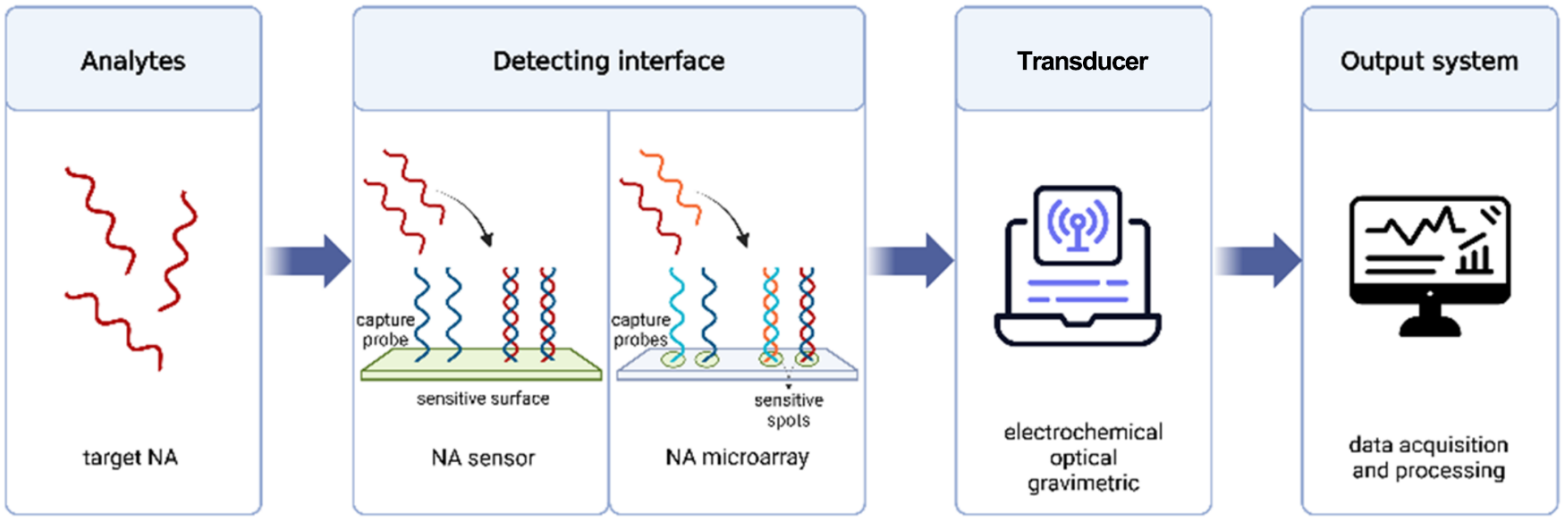
Schematic representation of the main components of NA biosensors and microarrays.

### 3.1 Development Process of NA Sensors and Microarrays

A key advantage of NA biosensors and microarrays over traditional PoCT is their potential reusability. These devices exploit the thermal stability of NAs, allowing probe regeneration through simple chemical or thermal methods without significant loss of hybridization efficiency. Lü et al.^162^ demonstrated this capability using biotinylated DNA probes, successfully regenerating their biosensor using either thermal treatment (brief heating at 70 °C for 2 min) or chemical treatment washing with 4 M urea for 2 min. After six regeneration cycles, binding efficiency decreased by only 20%, confirming these platforms’ reusability potential.

NA microarrays offer enhanced versatility through different probe immobilization technologies, including *in situ* synthesis and (contact and noncontact) printing methods (spotted microarrays). *In situ* synthesis directly creates ssNA probes on the microarray surface, providing reproducible manufacturing, standardized processes and analysis, and higher probe density (>1 × 10^6^ spots) than printed microarrays (1 × 10^4^ to 3 × 10^4^ spots). However, complex chemistry and high production costs make *in situ* synthesis less practical for custom development, requiring commercial manufacturing.^158^ Various companies have developed distinct DNA microarray technologies. Affymetrix^163^ uses mask-based photodeprotection, whereas Agilent^164^ employs inkjet technology with chemical deprotection.

On the other hand, spotted/printed microarrays use presynthesized probes applied through contact or noncontact printing. Contact printing microarray manufacturing use high-definition pins to deposit tiny probe volumes (between 100 pL and 2 μL) onto sensor surface.^165^ While offering flexibility for probe modification, they face significant challenges, including laborious clinical validation, complex reproducibility, quality control issues like nonuniform feature sizes, and variations in probe deposition across slides and batches.^161,165^ The labor-intensive probe design process and potential synthesis errors further complicate their use in diagnostic settings.^158^

Both NA biosensors and microarrays detect complementary ssNA sequences using optical, electrochemical, or gravimetric technologies.^51,126,158^ While biosensors have a simpler structure, microarrays offer high-throughput capabilities, enabling the simultaneous detection of thousands of gene expression patterns in a single experiment. Fluorescence dominates as the detection method, with systems like Agilent’s microarray^164^ using a two-color detection approach, labeling targets with different fluorescent dyes (Cy3 and Cy5) for comprehensive analysis.

### 3.2 Hybridization Control of NA Sensors and Microarrays

NA sensors and microarrays rely critically on the hybridization between surface-immobilized ssNA probes and cNA targets. The performance of these devices hinges on two paramount factors: selectivity and sensitivity. Selectivity enables precise identification of point mutations in the sample cNA, while sensitivity allows detection of low NA concentrations.^105^ High sensitivity is especially valuable as it can eliminate the need for sample amplification, thereby reducing diagnostic time and costs.

NA sensor and microarray hybridization processes often lack rigorous parameter control (Table 4), presenting significant methodological challenges. Most studies inadequately manage critical factors such as *T*_H_ (typically conducted at RT or 37 °C) without analyzing probes’ behavior; the hybridization time (ranging consistently from 5 min to O.N.); specificity assessment (frequently missing noncomplementary control probes, called scramble sequences). These inconsistencies can substantially impact sensor sensitivity and specificity. Microarrays have partially addressed these limitations through thermal control during hybridization, multiplexing capabilities, and design strategies to prevent cross-hybridization. However, despite improved hybridization control, NA microarray technologies still have significant drawbacks, including the requirement for specialized equipment (e.g., hybridization ovens, microarray readers). Additionally, most are currently research-oriented rather than diagnostic. The divergent performance between sensors and microarrays likely stems from their fundamentally different design approaches to NA detection.

**Table 4:** State of the Art of NA Biosensors and Microarrays

	Immobilization	Detection	Temperature	Time	Controls	Refs
biosensors	chemisorption	surface plasmon resonance	not disclosed	5 min	no	^48^
	chemisorption	electrochemical–labeled	not disclosed	30 min	yes, but not disclosed	^110^
	electrostatic immobilization	electrochemical–label-free	RT	up to 60 min	no	^166^
	covalent linking	photoluminescence	50 °C	4 h	no	^80^
	covalent linking	electrochemical–cyclic voltammetry and impedance	42 °C	20 min	yes, disclosed	^167^
microarrays	covalent linking	fluorescence	42 °C	O.N.	no	^61^
	covalent linking	fluorescence	45 °C	1 h	yes, disclosed	^60^
	photolithography	fluorescence	40–50 °C	16 h	-	^163^
	inkjet printing	fluorescence	65 °C	40 h	-	^164^

### 3.3 On the Path for PoCTs: Sensors and Microarrays

When considering cutting-edge diagnostic technologies, biosensors and microarrays stand out as important innovations. These electronic devices integrate transduction elements for signal transmission with biological recognition components.^11^ Though often labeled as PoCTs, they typically fall short of REASSURED criteria, requiring additional equipment, trained personnel, and laboratory settings for results acquisition and interpretation.^152^

This classification discrepancy stems partly from inconsistent regulatory frameworks. The BS EN ISO 15189:2022 defines PoCT as “examination performed near or at the site of a patient”, a definition that fails to incorporate the comprehensive WHO’ ASSURED criteria. Similarly, European regulations, including Directive 98/79/EC, cover these devices but provide insufficient guidance regarding self-testing applications. This regulatory gap highlights the urgent need for harmonized standards that accurately reflect the operational characteristics of emerging diagnostic technologies while establishing clear benchmarks for true PoC implementation.

## 4 Conclusions

The journey from molecular design to clinical reality for PoC for NA detection represents one of the most promising yet challenging frontiers in modern diagnostics. PoC technologies for NA detection offer promising potential to simultaneously reduce healthcare costs and improve patient care. At the molecular design level, the technologies currently used and under development for NA-based PoCT show significant progress, particularly in meeting the ASSURED criteria. However, despite these advances, substantial challenges remain in translating innovative research into practical, affordable diagnostic products that can be effectively implemented in healthcare systems.

The molecular foundation of these technologies has evolved significantly, with PoCT platforms advancing through progressive miniaturization, enhanced multiplexing capabilities, and enhanced connectivity frameworks that bridge the gap between laboratory-based molecular techniques and field-deployable diagnostic tools. This evolution offers unprecedented opportunities for decentralized diagnostics.

Nevertheless, the fundamental challenge remains bridging the gap between proof-of-concept prototypes and commercially viable products that meet rigorous standards for clinical reliability, manufacturing scalability, and economic feasibility. The transition from molecular design to practical implementation requires addressing the development of equipment-free detection methodologies that would substantially enhance accessibility and user-friendliness while significantly reducing both production costs and market entry barriers. In this light, the implementation of equipment-free systems, while beneficial, faces technical challenges, particularly regarding quantitative accuracy. Success in translating molecular innovations to clinical applications depends on several critical factors: NA immobilization techniques must achieve optimal probe density (around 10^12^ molecules/cm^2^); hybridization conditions, especially temperature, need precise control to maximize PoCT sensitivity and specificity; DoE methodologies are essential for systematically translating hybridization parameters from well-characterized solution-phase reactions to the more complex and less-understood solid-surface environments typical of biosensors. Given the profound impact of temperature on devices’ LOD, incorporating temperature control components (e.g., Peltier devices) during development stages may prove beneficial, even for technologies ultimately destined for equipment-free operation.

The path to clinical reality requires that meaningful clinical impact for NA-based PoCT extends beyond ASSURED criteria, necessitating robust data collection and management framework, sophisticated security protocols, along with comprehensive quality assurance systems. Moving from prototype to clinical implementation demands fully integrated systems that eliminate subjective interpretation and can significantly reduce errors while enhancing diagnostic reliability. Furthermore, establishing cost-effective manufacturing methods is crucial for making these technologies accessible, especially in low-resource or underdeveloped regions.

Innovations in molecular design are driving next-generation clinical solutions, focusing on advanced DNA nanostructures like DNA origami and tetrahedral assemblies. These structures enhance control over probe density and organization, addressing biosensor limitations. By combining computational modeling, automated DNA design software, and AI optimization, we can efficiently develop these platforms while tackling challenges such as temperature control and self-assembly. These advancements, alongside programmable DNA networks and universal manufacturing platforms, are essential for creating fully ASSURED-compliant NA-based PoCT systems.

The ultimate realization of clinical implementation requires successful integration of these technologies into clinical practice requires addressing standardization, validation, ethics, and patient access. Only through coordinated multidisciplinary collaboration among researchers, industry stakeholders, healthcare providers, and regulatory authorities can these complex barriers be effectively overcome.

The development of fully ASSURED NA-based PoCTs, combined with strong data protection and connectivity, could fundamentally transform molecular diagnostics. Successfully implementing this technology requires strong interdisciplinary collaborations to effectively navigate the complex relationships among molecular engineering, regulatory compliance, and healthcare implementation. This synergy is essential for fostering innovation and enhancing outcomes in the field. It has the potential to redefine personalized medicine by enabling earlier diagnoses and more timely, cost-effective, and universal disease management.

## Abbreviations

AI: artificial intelligence
AuNPs: gold nanoparticles
BSA: Bovine serum albumin
cRNA: complementary RNA
DNA: deoxyribonucleic acid
DoE: design of Experiment
EDC: 1-ethyl-3-(3-dimethylaminopropyl) carbodiimide
ELISA: enzyme-linked immunosorbent assay
FISH: fluorescence *in situ* hybridization
FITC: fluorescein isothiocyanate
GOPS: (3-Glycidyloxypropyl)­trimethoxysilane
HAD: helicase-dependent amplification
HIV: human immunodeficiency virus
HPV16: human papillomavirus Type 16
HRP: horseradish peroxidase
ICC: immunocytochemistry
IHC: immunohistochemistry
LAMP: loop-mediated isothermal amplification
LFA: lateral flow assay
LoC: lab-on-Chip technology
LOD: limit Of Detection
MBs: molecular beacons
MCH: mercaptohexanol
MedTechs: medical diagnostic technologies
miRNA: micro RNA
mRNA: messenger RNA
MutS: mutator S protein
MW: molecular weight
NA: nucleic acid
NASBA: nucleic Acid Sequence Based Amplification
NHS: *N*-hydroxysuccinimide
NPs: nanoparticles
nt: nucleotide
NW: nanowire
O.N.: over night
ON: oligonucleotide
PCR: polymerase chain reactions
PNA: peptide nucleic acid
PoC: point-of-care technology
PoCT: point-of-care testing
QCM: quartz crystal microbalance
RNA: ribonucleic acid
RPA: recombinase polymerase amplification
RT: room temperature
rs: Spearman’s rank correlation coefficient
SAM: self-assembled monolayer
SERS: surface-enhanced Raman scattering
SiMoT: single-molecule-with-a-large-transistor
ssDNA/RNA: single-stranded DNA/RNA
*T*_H_: hybridization temperature
*T*_m_: melting temperature
WHO: World Health Organization
